# Hyperoxia-activated circulating extracellular vesicles induce lung and brain injury in neonatal rats

**DOI:** 10.1038/s41598-021-87706-w

**Published:** 2021-04-22

**Authors:** Anum Ali, Ronald Zambrano, Matthew R. Duncan, Shaoyi Chen, Shihua Luo, Huijun Yuan, Pingping Chen, Merline Benny, Augusto Schmidt, Karen Young, Nadine Kerr, Juan Pablo de Rivero Vaccari, Robert W. Keane, W. Dalton Dietrich, Shu Wu

**Affiliations:** 1 Division of Neonatology and Batchelor Children’s Research Institute, Department of Pediatrics, University of Miami Miller School of Medicine, P. O. Box 016960, Miami, FL 33101 USA; 2 Department of Neurological Surgery, Miami Project to Cure Paralysis, University of Miami Miller School of Medicine, Miami, USA; 3 Department of Physiology and Biophysics, University of Miami Miller School of Medicine, Miami, FL USA

**Keywords:** Cell biology, Developmental biology, Molecular biology, Neuroscience, Diseases

## Abstract

Hyperoxia-induced lung injury plays a key role in the development of bronchopulmonary dysplasia (BPD), characterized by inflammatory injury and impaired lung development in preterm infants. Although BPD is a predictor of poor neurodevelopmental outcomes, currently it is uncertain how lung injury contributes to brain injury in preterm infants. Extracellular vesicles (EVs) are a heterogeneous group of cell-derived membranous structures that regulate intercellular and inter-organ communications. Gasdermin D (GSDMD) has emerged as a key executor of inflammasome-mediated cell death and inflammation. In this study, we utilized a neonatal rat model of BPD to assess if hyperoxia stimulates lung release of circulating EVs and if these EVs induce lung and brain injury. We found that hyperoxia-exposed rats had elevated numbers of plasma-derived EVs compared to rats maintained in room air. These EVs also had increased cargos of surfactant protein C, a marker of type II alveolar epithelial cells (AEC), and the active (p30) form of GSDMD. When these EVs were adoptively transferred into normal newborn rats via intravenous injection, they were taken up both by lung and brain tissues. Moreover, EVs from hyperoxic animals induced not only the pathological hallmarks of BPD, but also brain inflammatory injury in recipient rats, as well as inducing cell death in cultured pulmonary vascular endothelial cells and neural stem cells (NSC). Similarly, hyperoxia-exposed cultured AEC-like cells released EVs that also contained increased GSDMD-p30 and these EVs induced pyroptotic cell death in NSC. Overall, these data indicate that hyperoxia-activated circulating EVs mediate a lung to brain crosstalk resulting in brain injury and suggest a mechanism that links lung injury and neurodevelopmental impairment in BPD infants.

## Introduction

Extremely premature infants born at less than 28 weeks of gestational age (GA) are at great risk of having multi-organ injury and developmental abnormalities that predominately involve the lung and brain^1,2^. The lungs of these infants are immature at birth, which predisposes them to respiratory failure and in need of oxygen therapy^1,3^. However, life sustaining oxygen therapy can cause lung inflammation that leads to lung structure damage and ultimately to bronchopulmonary dysplasia (BPD)^1, 3^. The brains of these infants are also immature and are prone to injurious stimuli such as oxygen toxicity and inflammation. Consequently, these infants are at greater risk of developing both short-term and long-term neurological complications^2^. Thus, BPD survivors not only suffer from pulmonary dysfunction but are also often complicated with long-term neurodevelopmental impairment (NDI). There is mounting clinical evidence that even in the absence of catastrophic brain injuries, severe BPD is an independent risk factor for adverse neurodevelopmental outcomes^4–6^. However, in spite of recent advances in neonatal intensive care and extensive research, the extent to which BPD contributes to NDI is uncertain, and there is no effective therapy for either the prevention or treatment of these conditions.

Extracellular vesicles (EVs) are a heterogeneous group of cell-derived nano-sized membranous structures, including exosomes and microvesicles, that are increasingly recognized as signal mediators of intercellular as well as inter-organ communications, in health and disease^7,8^. EVs are released by a variety of living cells in response to inflammation, oxidative stress, and cell activation or damage and have been isolated from most bodily fluids including bronchoalveolar lavage fluid (BALF)^9^ and blood and cerebrospinal fluid (CSF)^10^. EVs carry complex cargos of proteins, lipids and nucleic acids, and their cargo composition is highly dependent on the biological function of the parental cells^7–10^. Being membranous, EVs protect their cargo from the extracellular environment, thus allowing for safe transport and delivery of their intact cargo to near or distant target cells, resulting in modification of the target cell’s gene expression, signaling pathways and overall function^7–10^. Recently, lung epithelial cell-secreted EVs isolated from BALF have been shown to play a role in regulating inflammatory responses in adult lung diseases^11,12^. In preterm infants with severe BPD, increased numbers of EVs were detected in their tracheal aspirates, and interestingly, the majority of these EVs were found to be of epithelial origin^13^. These data suggest that lung epithelial cells can release bioactive EVs into airspace fluid upon inflammatory injury. Similarly, EVs contribute to a number of adult central nervous system disorders, and they can bidirectionally cross the blood brain barrier (BBB)^14,15^. Moreover, we have recently reported that traumatic brain injury in adult patients and mice causes the release of EVs into the circulation that travel to the lung to cause acute lung injury, confirming EV BBB transit and suggesting a brain to lung crosstalk^16^. However, to date, there are no clinical or pre-clinical studies that report lung epithelial cells releasing EVs into the circulation that can cross the BBB and induce injury in the developing brain.

Gasdermin D (GSDMD), a 53-kilodalton (kDa) cytosolic protein, was recently found to be a key executer of pyroptosis, a form of inflammasome-mediated cell death^17–19^. Inflammasome activation by pathogens or host-derived danger signals leads to the activation of caspase-1 and caspase-1-mediated cleavage of GSDMD releases the GSDMD’s 30-kDa N-terminal domain (p30) which oligomerizes in the cell membrane to form pores causing cellular swelling, membrane rupture and cell death^17–19^. The pores additionally allow rapid release of active interleukin (IL)-1β and IL-18, resulting in secondary inflammation^17–19^. We have previously demonstrated a critical role for the inflammasome pathway in experimental models of BPD^20–22^, and importantly, inhibition of GSDMD activation attenuates hyperoxia-induced lung and brain injury^22^. Moreover, GSDMD-p30 has also recently been detected in serum exosomal microparticles from adult patients with sepsis and acute lung injury^23^, but it is presently unknown if exosomal GSDMD plays a mechanistic role in the pathogenesis of BPD or brain injury in preterm infants.

In the present study, we tested the hypothesis that hyperoxia-induced neonatal lung and brain injury is mediated by circulating GSDMD-laden EVs released by alveolar type II epithelial cells (AEC) after their uptake by lung and brain tissues. We found supporting evidence for this hypothesis using a combination of in vivo adoptive EV transfer experiments in neonatal rats and in vitro studies using cultured AEC, pulmonary vascular endothelial cells (PVEC) and neural stem cells (NSC).

## Results

### Hyperoxia exposure stimulates the release of EVs with an increased cargo of SPC/GSDMD into the circulation of neonatal rats

**Figure 1:**
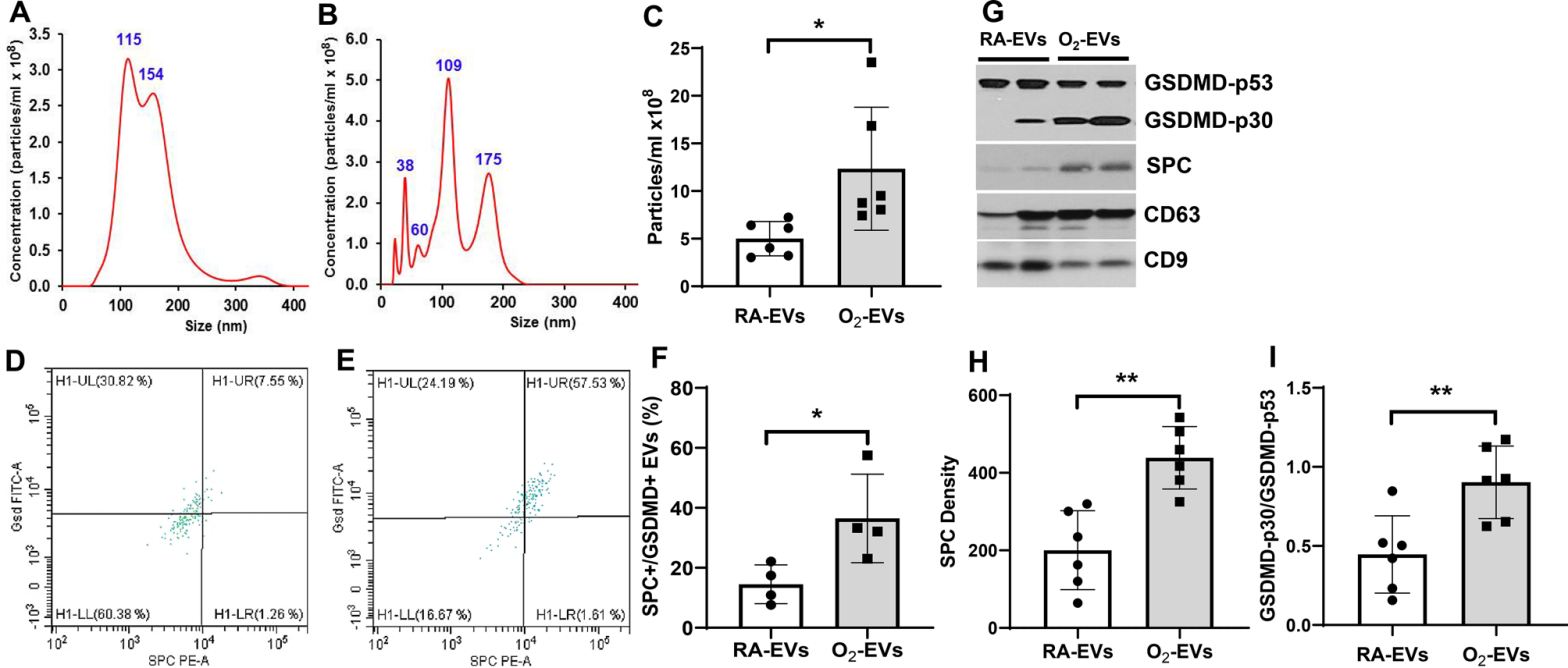
Hyperoxia stimulates the release of EVs with an increased cargo of SPC/GSDMD into the circulation of neonatal rats. Newborn rats on postnatal day 1 (P1) were exposed to room air (RA) or hyperoxia, 95% O_2_ (O_2_) for 14 days and EVs were isolated from the plasmas of these animals. Nanosight tracking assay showed that both room air-exposed EVs (RA-EVs, (**A**)) and hyperoxia-exposed EVs (O_2_-EVs, (**B**)) contained particles that were prodominately 100–150 nm in diameter, but O_2_-EVs had increased particle concentrations as compared to RA-EVs ((**C**), n = 6/group, *P < 0.05). FACS analysis demonstrated that RA-EVs (**D**) had a smaller population of SPC + /GSDMD + EVs compared to O_2_-EVs (**E**) ((**F**), n = 4/group, *P < 0.05). (**G**) Representative Western blots of SPC, GSDMD, CD9 and CD63. O_2_-EVs had increased cargos of SPC (**H**) and GSDMD-p30 (**I**) compared to RA-EVs (n = 6/group, **P < 0.01).

To determine if hyperoxia stimulates lung epithelial cells to release GSDMD-containing EVs into the circulation, we analyzed EV isolates from the plasma of neonatal rats maintained in room air (RA-EVs) or hyperoxia (O_2_-EVs) for 14 days. Nanosight tracking assay revealed that both RA-EVs (Fig. 1A) and O_2_-EVs (Fig. 1B) contained nanoparticles that were primarily of a 30–150 nm exosome size, with both peaking at 100–150 nm in diameter, but the nanoparticle concentrations of O_2_-EVs were 2.4-fold higher than RA-EVs (Fig. 1C, *P* < 0.05). FACS analysis after capture on anti-tetraspanin (CD9, CD63 and CD81) beads confirmed the exosome nature of the EVs^7, 8^ and demonstrated that the RA-EVs (Fig. 1D) contained a smaller population of SPC + /GSDMD + EVs compared to O_2_-EVs (Fig. 1E) (Fig. 1F, RA-EVs 14.5 ± 6.5% vs. O_2_-EVs 36.5 ± 14.8%, *P* < 0.05). Western blot analysis confirmed that O_2_-EVs contained twofold increased levels of SPC (Fig. 1G, H, O2-EVs 438.7 ± 80.5 vs. RA-EVs 200.3 ± 101.8, *P* < 0.01) and activated GSDMD-p30 compared to RA-EVs, which contained primarily inactive GSDMD-p53 (Fig. 1G, I, O2-EVs 0.90 ± 0.23 vs. RA-EVs 0.45 ± 0.24, *P* < 0.01) (Supplemental Fig. 1G). Western blot analysis also confirmed that both RA-EVs and O_2_-EVs contained relatively equal amounts of exosomal markers, CD63 and CD9 tetraspanins, when the proteins were loaded in equal amounts/sample (Fig. 1G). Thus, hyperoxia stimulates AEC to release circulatory EVs, with an increased cargo of active GSDMD which are characteristically exosomes based on their size and high levels of multiple tetraspanin expression, although the presence of other nano or microparticles with similar characteristics cannot be ruled out.

### Adoptively transferred circulating EVs travel to the lung and brain of normal neonatal rats

**Figure 2:**
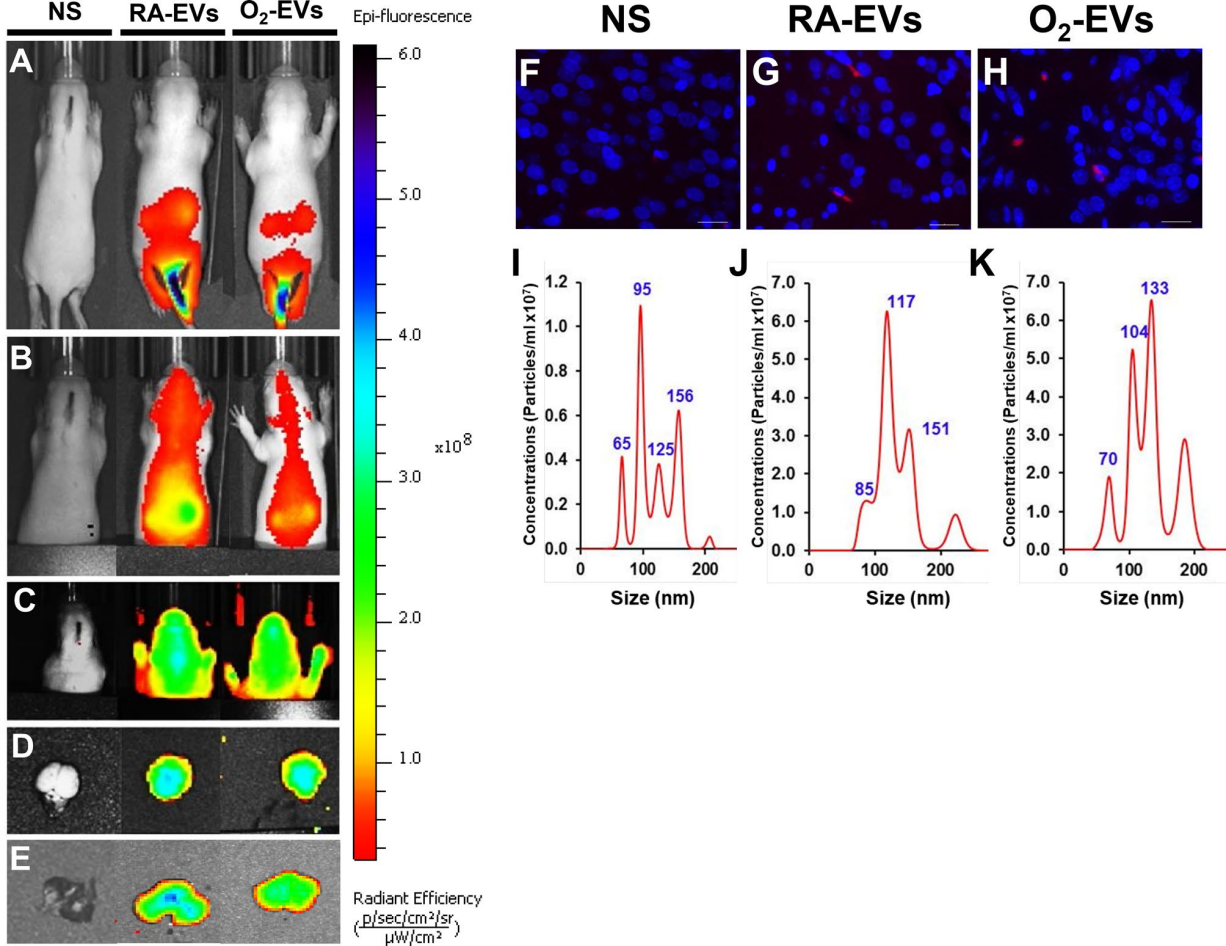
Adoptively transferred circulating EVs track to the lung and brain of normal neonatal rats. Exo-glow dye sham-labeled normal saline (NS), labeled RA-EVs or O_2_-EVs was injected via tail vein into normal neonatal rats at P7. In vivo imaging was acquired at 15 min (**A**), 1 h (**B**) and 4 h (**C**). Brains (**D**) and lungs (**E**) were dissected at 4 h for ex vivo imaging. In vivo near IR signals were detected in the brain and lung regions at 1 h and 4 h and also ex vivo in dissected brain and lung tissues at 4 h. Dil sham-labeled NS, labeled RA-EVs or O_2_-EVs were injected via tail vein into normal neonatal rats at P7 and brain tissues and CSF samples were collected 24 h later. Brain sections from NS injected rats (**F**) were negative but both RA-EVs injected (**G**) and O_2_-EVs (**H**) injected animals showed Dil signals (red flourecence). Nanosight tracking of EVs isolated from the CSF showed increased EV particle concentrations in CSF from RA-EVs (**J**) and O_2_-EVs (**K**) injected rats compared to NS injected rats (**I**). Scale bar: 50 µm.

As a prelude to determining if circulating EVs from hyperoxia-exposed rats can cause lung and brain injury, we performed adoptive transfer experiments and examined their potential to traffic not only to the lung but also to the brain. RA-EVs or O_2_-EVs were labeled with tracking dyes and adoptively transferred into normal neonatal rats by intravenous injection. As illustrated in Fig. 2A–C, both Exo-Glow labelled RA-EVs and O_2_-EVs rapidly distributed throughout the body and were localized in the lungs and brain at 1 and 4 h after tail vein injection. Their uptake by the brain (Fig. 2D) and lung (Fig. 2E) tissues was further confirmed by ex vivo imaging at 4 h. To determine if they are present in brain tissue for longer than 4 h, we similarly injected Dil-dye-labeled EVs and isolated EVs from the CSF and examined brain tissue sections 24 h later. We detected fluorescent Dil signals in the brain tissue sections from both RA-EVs and O_2_-EVs injected rats but not from sham animals (Fig. 2F–H), and when compared to sham animals (Fig. 2I, 4.95 ± 0.57 × 10^7^, n = 4 pooled of 3 CSF) animals that received either RA-EVs (Fig. 2J, 17.55 ± 1.9 × 10^7^, n = 2 pooled of 3 CSF, *P* < 0.01) or O_2_-EVs (Fig. 2K, 26.7 ± 16.6 × 10^7^, n = 2 pooled of 3 CSF, *P* < 0.05) had much higher concentrations of CSF EVs. Overall, these results confirm that hyperoxia-induced circulating EVs can cross the BBB and be taken up by brain cells.

### Adoptive transfer of circulating EVs from hyperoxia exposed rats induces lung inflammatory injury in normal neonatal rats

**Figure 3:**
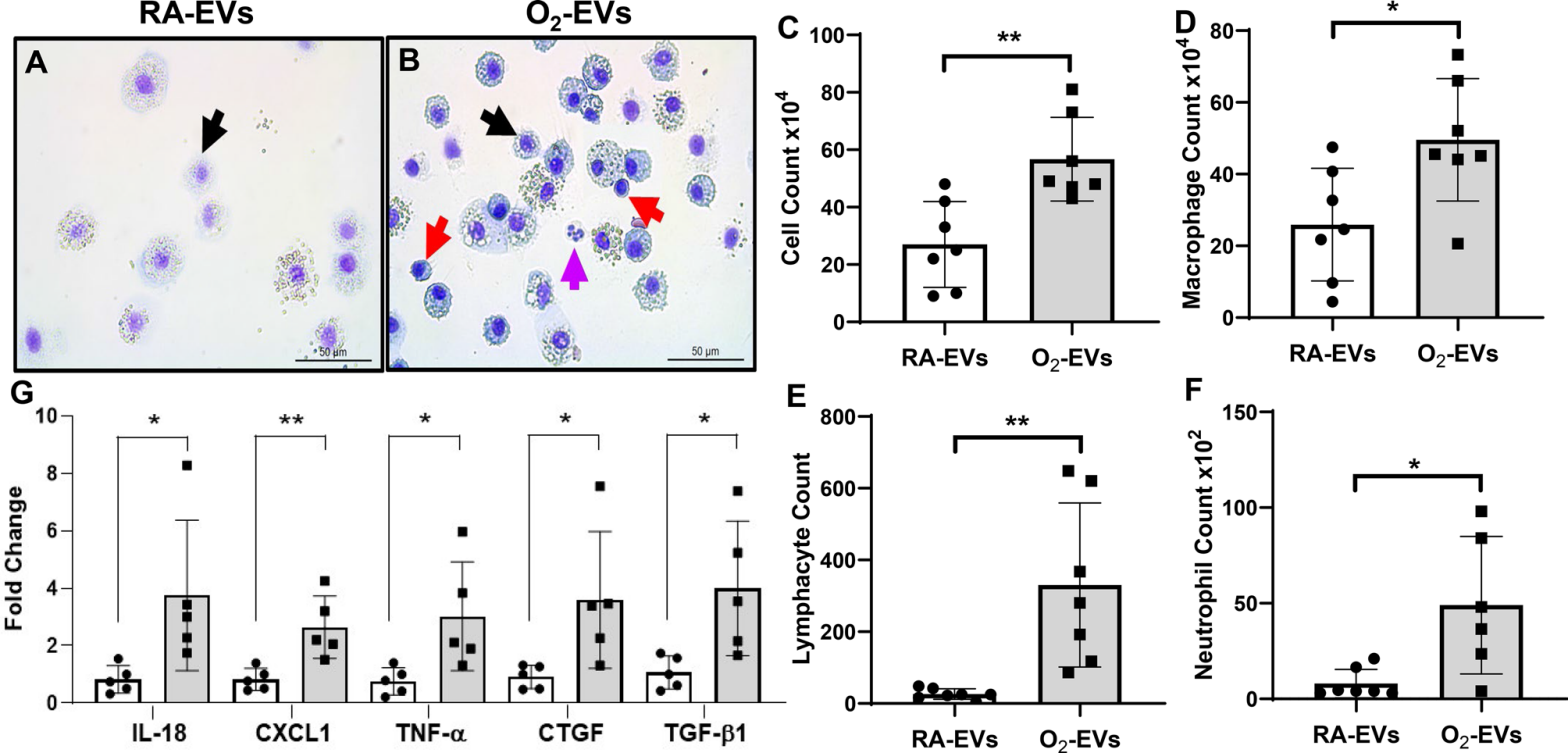
Circulating EVs from hyperoxia-exposed rats induce lung inflammation when adoptively transferred into normal neonatal rats. RA-EVs and O_2_-EVs were injected into normal neonatal rats via temple vein on P3 and tail vein on P7. Representative cytospins of bronchoalveolar lavage fluids (BALF) from RA-EVs injected (**A**) and O_2_-EVs injected (**B**) rats. The total cell count ((**C**), **P < 0.01), macrophage count ((**D**), black arrow, *P < 0.05), lymphocyte count ((**E**), red arrow, **P < 0.01) and neutrophil count ((**F**), purple arrow, *P < 0.05) were increased in O_2_-EVs injected rats. n = 7/group. (**G**) qRT-PCR demonstrated that lung tissues from O_2_-EVs injected rats have increased gene expression of IL-18, CXCL1, TNF-α, CTGF and TGF-β compared to RA-EVs injected rats (n = 5/group, *P < 0.05 and **P < 0.01).

We next investigated if adoptive transfer of circulating EVs with increased cargo of GSDMD-p30 could cause inflammatory lung injury in normal neonatal rats. We found that when compared to RA-EVs injected rats (Fig. 3A), rats that received O_2_-EVs (Fig. 3B) had increased numbers of total leukocytes in their BALF (Fig. 3C, O2-EVs 57.7 ± 14.6 × 10^4^ vs. RA-EVs 27.0 ± 13.8 × 10^4^, *P* < 0.01). Differential counts of macrophages, lymphocytes, and neutrophils were similarly elevated in rats receiving O_2_-EVs when compared to rats receiving RA-EVs, accordingly, macrophages (Fig. 3D, O2-EVs 49.6 ± 17.1 × 10^4^ vs. RA-EVs 25.9 ± 14.5 × 10^4^, *P* < 0.05); lymphocytes (Fig. 3E, O2-EVs 330.2 ± 228.5 vs. RA-EVs 26.1 ± 13.7, *P* < 0.01); and neutrophils (Fig. 3F, O2-EVs 4456.1 ± 383.7 vs. RA- EVs 385.7 ± 312.9, *P* < 0.05). We further analyzed lung inflammation by measuring lung tissue gene expression of several inflammation-associated factors by qRT-PCR (Fig. 3G). The lungs of rats that received O_2_-EVs had increased expression of the inflammatory mediators IL-18 (*P* < 0.05), TNF-α (*P* < 0.05), chemokine CXCL1 (*P* < 0.01), and profibrotic factors CTGF (*P* < 0.05) and TGF-β (*P* < 0.05). Thus, O_2_-EVs induce lung inflammatory injury.

### Circulating EVs from hyperoxic rats inhibit alveolarization and vascular development in normal neonatal rats in vivo and decrease cell proliferation as well as increase cell death in PVEC in vitro

**Figure 4:**
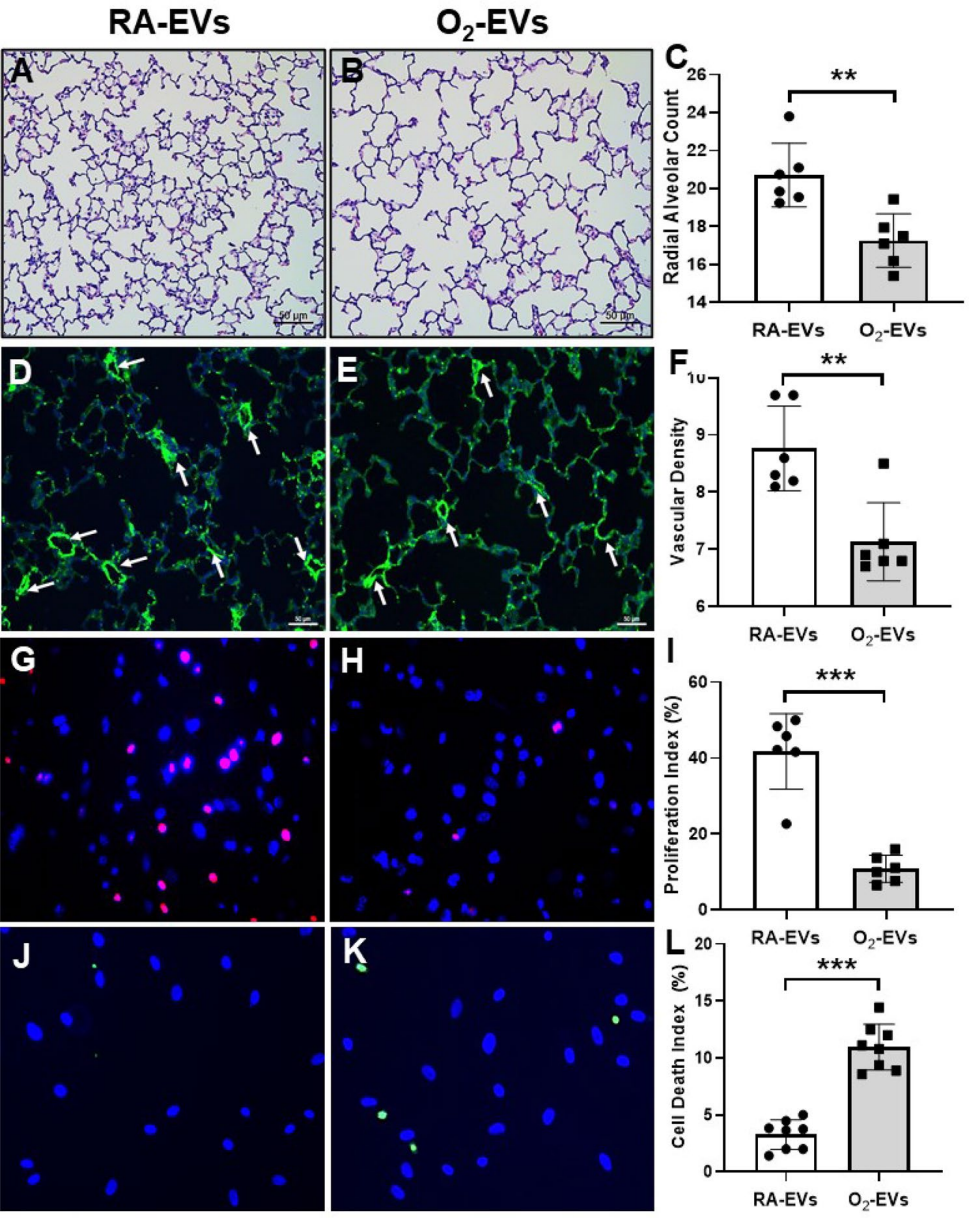
Circulating EVs from hyperoxia-exposed rats inhibit alveolar and vascular development in vivo and decrease cell proliferation and increase cell death in PVEC in vitro. H&E staining showed normal alveolar development in RA-EVs injected rats (**A**), but O_2_-EVs injected rats had larger and simplified alveolar structure (**B**). Scale bar: 50 µm. Morphometric analysis showed there was a decreased radial alveolar count (RAC) in O_2_-EVs injected rats as compared to RA-EVs injected rats ((**C**), n = 6/group, **P < 0.01). Immunofluorescence for vWF (an endothelial marker, green signals) and DAPI nuclear stain (blue signals) of lung sections from RA-EVs injected rats (**D**) and O_2_-EVs injected rats (**E**). Vascular densities (vessels < 50 µm in diameter, white arrows) were decreased in O_2_-EVs injected rats compared to RA-EVs injected rats ((**F**), n = 6/group, **P < 0.01). Scale bar: 50 µm. PVEC were cultured in the presence of RA-EVs or O_2_-EVs for 48 h. (**G**) and (**H**) Ki67 staining (pink signals) and DAPI nuclear staining (blue signals) showed decreased cell proliferation in O_2_-EVs treated PVEC compared to RA-EVs treatment ((**I**), n = 6/group, ***P < 0.001). (**J**) and (**K**) TUNEL (green signals) and DAPI nuclear staining (blue signals) showed increased cell death in O_2_-EVs treated PVEC compared to RA-EVs treatment ((**L**), n = 8/group, ***P < 0.001).

Impaired alveolarization and vascular development are hallmarks of BPD. Thus, we next evaluated alveolar and vascular development in neonatal rats that received adoptive transfer of RA-EVs and O_2_-EVs. Microscopy of H&E stained lung tissue sections showed that rats injected with O_2_-EVs had larger and more simplified alveolar structures as well as significantly decreased radial alveolar count (RAC) in comparison to rats that received RA-EVs (Fig. 4A–C, O2-EVs 17.6 ± 1.4 vs. RA-EVs 20.7 ± 1.7, *P* < 0.01). Moreover, vascular density was significantly decreased in rats injected with O_2_-EVs (Fig. 4D–F, O2-EVs 7.1 ± 0.7 vs. RA- EVs 8.8 ± 0.7, *P* < 0.01). These results confirm that hyperoxia causes the release of EVs capable of inducing BPD-like pathology.

To further examine the effects of circulating EVs on lung vasculature, we treated cultures of PVEC with RA-EVs and O_2_-EVs and evaluated cell proliferation by Ki67 immunofluorescent staining and cell death by TUNEL. We found that PVEC treated with O_2_-EVs had markedly decreased proliferation indices compared to PVEC treated with RA-EVs (Fig. 4G–I, O2-EVs 10.8 ± 3.6% vs. RA-EVs 41.8 ± 9.9%, *P* < 0.001). This inhibitory effect can at least be partially attributed to increased cell death as we also observed significantly more TUNEL positive cells in PVEC cultures treated with O_2_-EVs than with RA-EVs (Fig. 4J–L, O2-EVs 10.9 ± 2.0% vs. RA-EVs 3.3 ± 1.3%, *P* < 0.001). Thus O_2_-EVs exhibit inhibitory effects on the growth of lung vasculature in vivo and in vitro.

### Adoptively transferred circulating EVs from hyperoxia exposed rats induce brain inflammatory injury in normal neonatal rats

**Figure 5:**
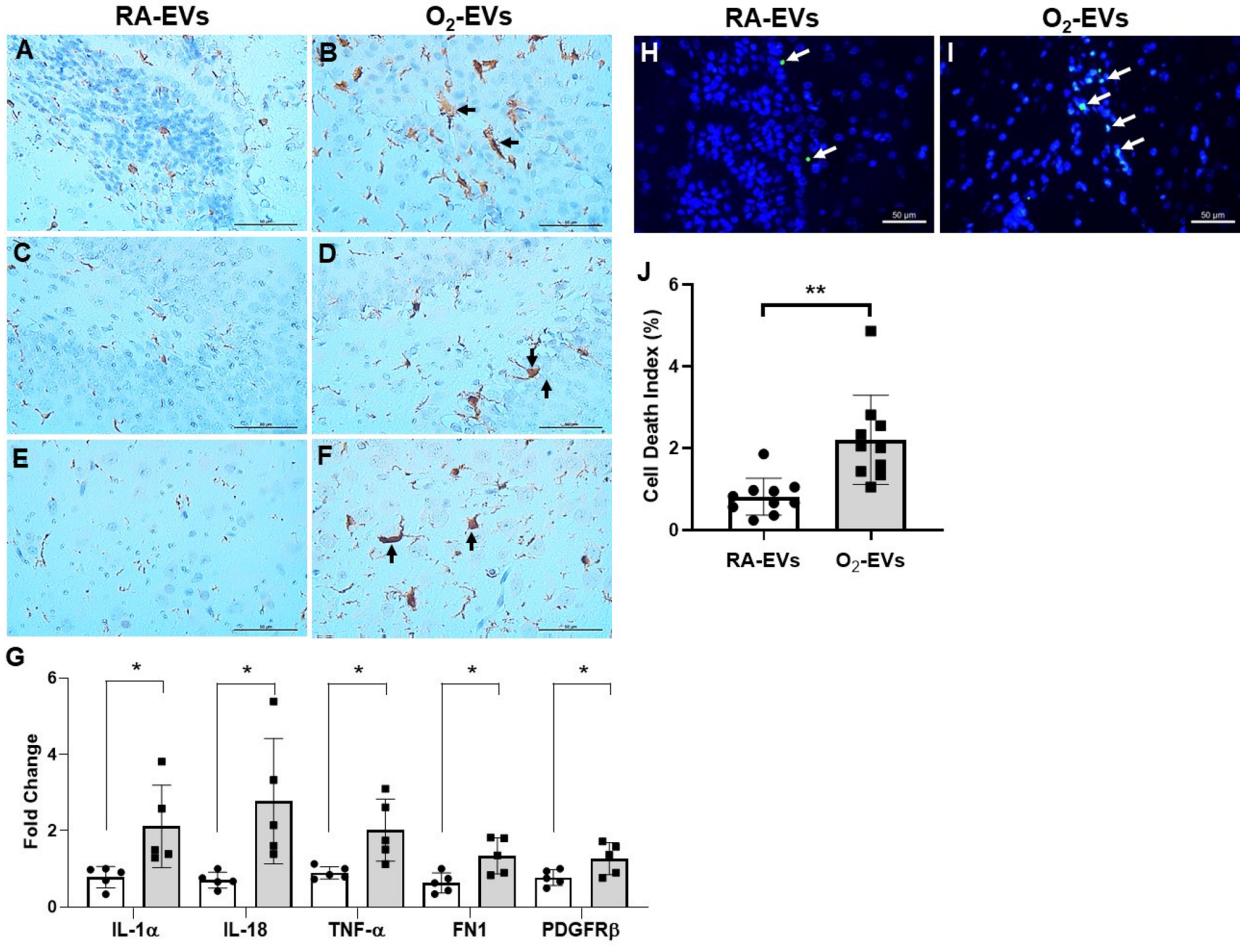
Circulating EVs from hyperoxia-exposed rats induce brain inflammatory injury when adoptively transferred into normal neonatal rats. (**A**) and (**B**): SVZ. (**C**) and (**D**): SGZ. (**E**) and (**F**): Cortex. Immunostaining for AIF-1, a microglia marker (brown signals) demonstrated enlarged activated microglial cells (black arrows) in the SVZ (**B**), SGZ (**D**) and cortex (**F**) of rats receiving O_2_- EVs injection. Scale bar: 50 µm. qRT-PCR showed increased gene expression of IL-1α, IL-18, TNF-α, fibronectin (FN1) and platelet derived growth factor receptor beta (PDGFRβ) in brain tissues of O_2_-EVs injected rats compared to RA-EVs injected rats ((**G**), n = 5/group, *P < 0.05). When compared to RA-EVs injected rats (**H**), the TUNEL assay showed increased dead cells (white arrows) in the SVZ of O_2_-EVs injected rats (**I**) ((**J**), n = 10/group, **P < 0.01). Scale bar: 50 µm.

We further investigated if circulating EVs from hyperoxia-exposed rats can also cause brain inflammatory injury when adoptively transferred into normal neonatal rats. First, we performed immunostaining for allograft-inflammatory-factor-1 (AIF-1), a marker for microglial cells, on brain tissue sections and found that when compared to RA-EVs, O_2_-EVs greatly increased the presence of enlarged activated inflammatory microglial cells in the SVZ (Fig. 5A, B), SGZ (Fig. 5C, D) and cortex (Fig. 5E, F) of recipient rats. We also performed qRT-PCR to assess gene expression of inflammatory mediators involved in microglial cell activation, namely: IL-1α, IL-18, TNF-α, fibronectin (FN1) and platelet derived growth factor receptor beta (PDGFRβ), and we found that all of these factors were significantly higher in rats that received O_2_-EVs when compared to rats that received RA-EVs (Fig. 5G, *P* < 0.05). Moreover, when we further examined the brain sections for evidence of neural cell death by TUNEL assay we found a 3.3 fold increase in dead cells in the SVZ of rats who received O_2_-EVs , compared to rats who received RA-EVs (Fig. 5H–J, O2-EVs 2.7 ± 1.29% vs. RA-EVs 0.8 ± 0.45%, *P* < 0.01). These novel findings indicate that O_2_-EVs can cause brain damage.

### Circulating EVs from hyperoxia exposed rats decrease NSC proliferation and increase cell death in vitro

**Figure 6:**
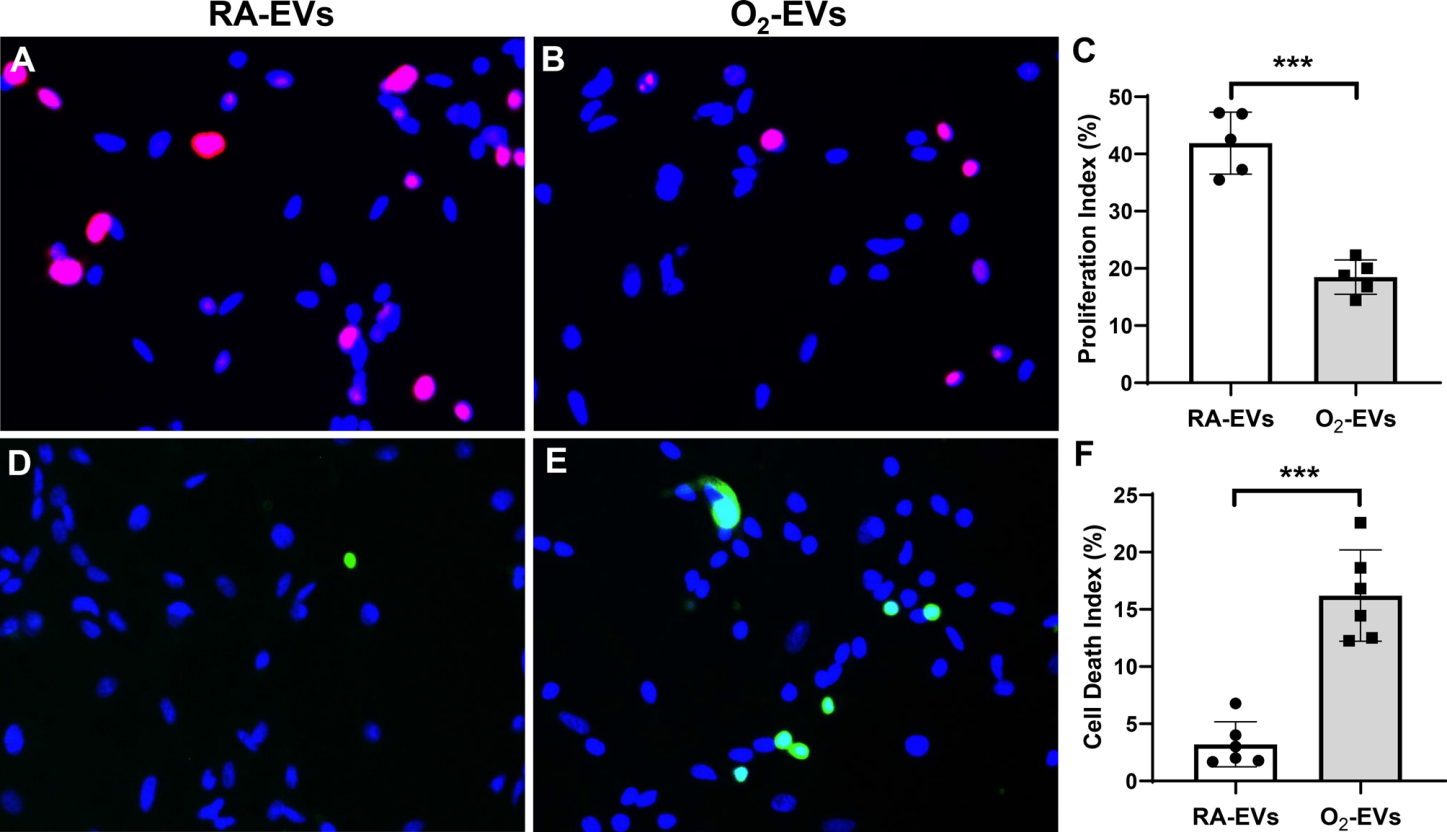
Circulating EVs from hyperoxia-exposed rats decrease cell proliferation and increase cell death in NSC in vitro. NSC were cultured in the presence of RA-EVs or O_2_-EVs from rats for 48 h. (**A**) and (**B**): Ki67 staining (pink signals) and DAPI nuclear staining (blue signals) showed decreased cell proliferation in O_2_-EVs treated cells compared to RA-EVs treated cells ((**C**), n = 5/group, ***P < 0.001). (**D**) and (**E**): TUNEL (green signals) and DAPI nuclear staining (blue signals) showed increased cell death in O_2_-EVs cultured cells compared to RA-EVs cultured cells ((**F**) n = 6/group, ***P < 0.001).

To examine the effects circulating EVs had on the brain at a cellular level, we treated cultures of NSC with RA-EVs and O_2_-EVs and found that O_2_-EVs decreased NSC proliferation by more than two-fold compared to RA-EVs (Fig. 6A–C, O2-EVs 18.5 ± 3.0% vs. RA-EVs 41.9 ± 5.4%, *P* < 0.001). Furthermore, the observed growth inhibition may be mediated by cell death since NSC had increased cell death indices when cultured with O_2_-EVs, compared to cells cultured with RA- EVs (Fig. 6D–F, O2-EVs 16.2 ± 3.9% vs. RA-EVs 3.2 ± 1.9%, *P* < 0.001). These results suggest that the O_2_-EVs induced brain cell death observed in our in vivo studies may be primarily occurring in NSC.

### Hyperoxia stimulates MLE-12 cells to release EVs with an increased cargo of GSDMD-p30 which induce NSC cell death in vitro

**Figure 7:**
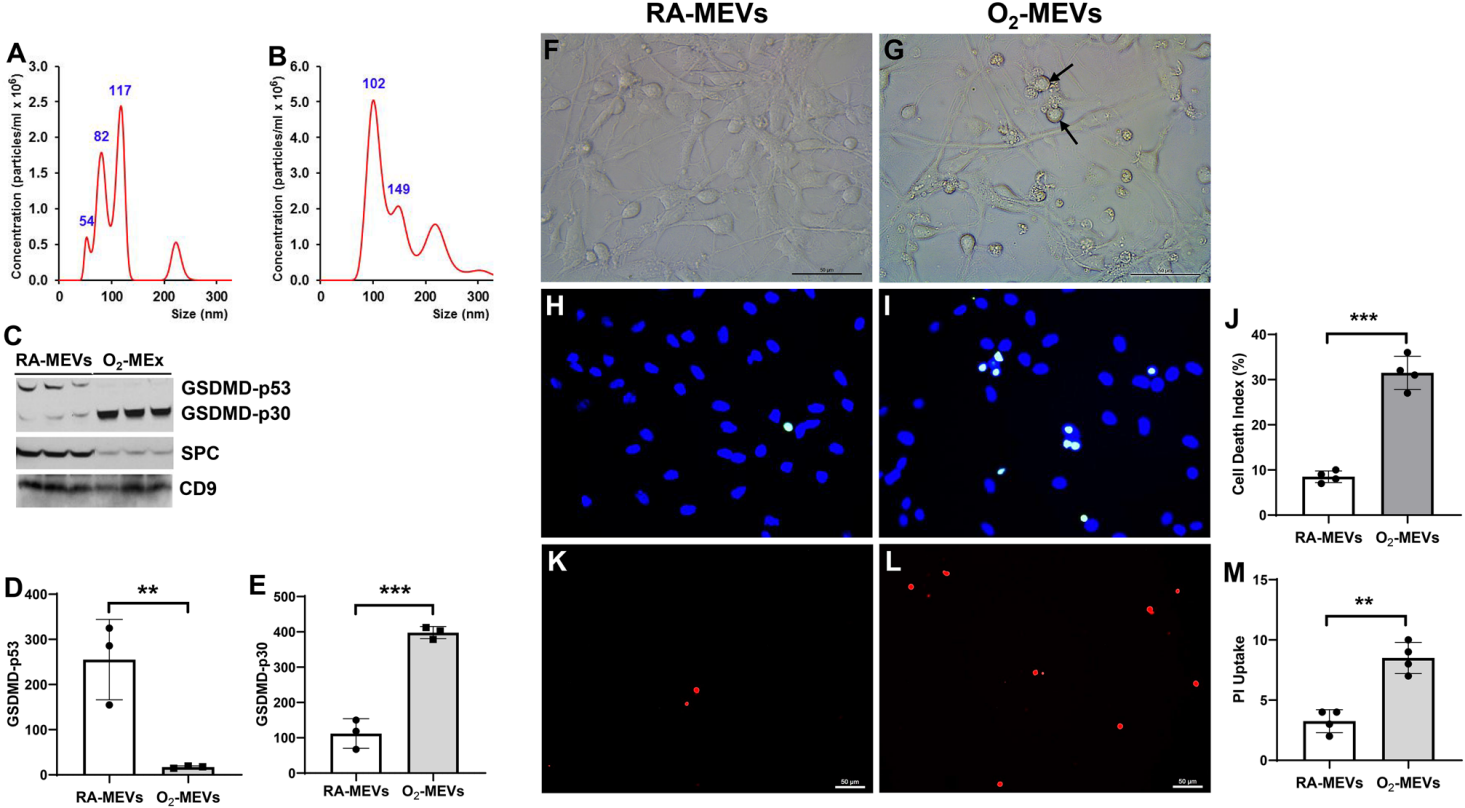
Hyperoxia stimulates MLE-12 cells to release EVs with an increased cargo of GSDMD-p30 that induce NSC death in vitro. MLE-12 cells were cultured under RA or 95% O_2_ for 48 h. EVs in the culture media were isolated and analyzed by nanosight tracking, (**A**) EVs from RA-maintained MLE-12 cells (RA-MEVs) and (**B**) EVs from O_2_-exposed MLE-12 cells (O_2_-MEVs). (**C**) Representative western blots of GSDMD, SPC and CD9 (Supplemental Fig. 7C). O_2_-MEVs had decreased GSDMD-p53 cargos ((**D**), **P < 0.01) and increased GSDMD-p30 cargos ((**E**), ***P < 0.001) compared to RA- MEVs. n = 3/group. NSC were cultured in the presence of RA-MEVs (**F**, **H**, **K**) or O_2_-MEVs (**G**, **I**, **L**) for 48 h. (**F**) and (**G**): live cell imaging under phase contrast showed blebbed cells (black arrow) in O_2_-MEVs treatment. (**H**) and (**I**): TUNEL (green signals) and DAPI nuclear staining (blue signals) showed increased cell death in O_2_-MEVs treated NSC compared to RA-MEVs treated NSC ((**J**) n = 4/group, ***P < 0.001). (**K**) and (**L**): PI uptake (red signals) demonstrated increased pyroptotic cell death with O_2_-MEVs treatment compared to RA-MEVs treatment ((**M**) n = 4/group, ***P < 0.001). Scale bar: 50 µm.

In order to confirm our in vivo finding that hyperoxia stimulated AEC to release EVs that contain an increased cargo of activated GSDMD, we examined the effect of hyperoxia on EV release from cultured MLE-12 cells, an AEC-like cell line. We found EVs isolated from the supernatant media of cells cultured under room air (RA-MEVs) or hyperoxia (O_2_-MEVs) conditions were comparably sized, with peak distributions of 100–150 nm, which is similar to our neonatal rat plasma EVs (Fig. 7A, B), but in contrast to our in vivo results, RA-MEVs and O_2_-MEVs had similar EV concentrations (data not shown). However, like circulating EVs induced by hyperoxia, O_2_-MEVs when compared to RA-MEVs had minimal full-length GSDMD-p53 (Fig. 7C, D, O2-MEVs 17.3 ± 2.5 vs. RA-MEVs 255.2 ± 89.0, *P* < 0.01) but contained an increase cargo of activated GSDMD-p30 (Fig. 7C, E, O2-MEVs 397.8 ± 17.4 vs. RA-MEVs 111.9 ± 41.8, *P* < 0.001), and both RA-MEVs and O_2_-MEVs contained SPC as well as EV exosomal marker CD9 (Fig. 7C), confirming their AEC lineage and EV nature. These results reinforce our in vivo conclusion that hyperoxia activation of AEC GSDMD results in AEC release of GSDMD-p30 rich EVs into the circulation.

To determine if O_2_-MEVs also induced increased NSC inflammasome-mediated cell death, as rat O_2_-EVs had, we treated NSC with MLE-12 cell EVs. We found this to be the case, as when compared to RA-MEVs exposed cells (Fig. 7F), O_2_-MEVs increased pyroptotic cell death as observed appearance of “blebbed” cells (Fig. 7G), and as measured an increased number of TUNEL positive cells (Fig. 7H–J, O_2_-MEVs 32.3 ± 2.7% vs. RA-MEVs 9.3 ± 3.7%, *P* < 0.001) and an increased number of propidium iodide (PI) positive cells (Fig. 7K–M, O_2_-MEVs 7.25 ± 1.25 vs. RA-MEVs 3.25 ± 0.95, *P* < 0.01). These results suggest that circulatory GSDMD-rich EVs are responsible for in vivo neural cell death that leads to brain injury in hyperoxia-exposed rodents.

## Discussion

BPD, characterized by inflammatory injury and impaired lung development, is the most common morbidity complicating preterm birth^1,3^, and a predictor of poor neurodevelopmental outcomes^4–6^. Currently, it is unclear to what extent lung injury contributes to brain injury in preterm infants, and there are no effective therapies for these two conditions. EVs have recently emerged as key regulators of cellular crosstalk by shuttling functional and signaling molecules to both neighboring and distal cells, affecting physiological functions or pathological responses of the recipient cells^7,8^. Our previous studies have shown that the inflammasome-GSDMD cascade plays a critical role in a hyperoxia-induced mouse model of BPD and brain injury^22^. In the present study, we have isolated nanoparticles from the plasma of neonatal rats that are considered to be EVs by MISEV2018 standards^7^ due to their extracellular nature, isolation method (commercial polymer precipitation kit), nanoparticle size, lipid membrane structure (Dil-dye labelling), general protein and non-lipid content (BCA assay and ExoGlow-Vivo-EV labelling), and the presence of EV tetraspanin membrane protein markers characteristic of exosomes by both Western blot (CD9 and CD63) and capture by anti-tetraspanin (CD9, CD63, and CD81)-conjugated magnetic beads prior to FACS analysis. Moreover, we demonstrated that hyperoxia-exposed neonatal rats had elevated levels of plasma EVs containing an increased cargo of both the AEC marker, SPC, and GSDMD-p30 compared to room air-maintained controls. We also found that these EVs traveled not only to the lung but also to the brain when adoptively transferred into the peripheral circulation of normal newborn rats. Importantly, adoptive transfer of O_2_-EVs induced not only the pathological hallmarks of BPD but also led to brain inflammatory injury. We further showed that O_2_-EVs induced cell death in cultured PVEC and NSC. We also confirmed in vitro that hyperoxia stimulated AEC to release GSDMD-p30-containing EVs into their culture media, and these EVs induced NSC pyroptotic death. Overall, these findings support a novel mechanism through which hyperoxia-activated circulating EVs induces lung and brain injury and suggest that targeting this mechanism may have therapeutic value in preventing BPD-associated brain injury. Moreover, if elevated levels of circulating SPC+/GSDMD+ EVs also occur in preterm infants, they could be used as a predictive biomarker of BPD and possibly poor neurodevelopmental outcome.

One of the key findings of this study is the discovery of an increased number of circulatory AEC-derived EVs in hyperoxia-exposed neonatal rats. While there are previously published studies showing the presence of hyperoxia-induced EVs in BALF and serum of adult rodents, this report is the first linking circulating AEC-derived EVs to a hyperoxia-induced neonatal rat model of BPD. For example, two previous studies in adult rodent models of hyperoxia-induced acute lung injury have shown that lung epithelial cells can release EVs and microvesicles into BALF or serum^11,24^. Another recent study found tracheal aspirates from infants with severe BPD had increased numbers of, but smaller, exosomes compared with term controls^13^. This study also found increased exosomes in BALF from a hyperoxia-induced mouse model of BPD. Interestingly, the majority of these exosomes were epithelial in origin, but this study did not evaluate whether there were increased plasma exosomes in either the clinical or experimental BPD arm of the study. Thus, our study extends this report to show that hyperoxia stimulates AEC to release EVs with some exosomal characteristics into the circulation using a rat BPD model. Additionally it also suggests that if elevated levels of SPC-positive AEC-derived EVs could be used as a novel biomarker of BPD, they would be easily obtainable from plasma as opposed to invasive BALF sampling.

Our results also demonstrated that the circulating EVs from hyperoxia-exposed neonatal rats had increased cargos of GSDMD, particularly its pyroptosis-inducing inflammasome-activated GSDMD-p30 form. Our FACS data further showed that the increased GSDMD was primarily present in SPC+/GSDMD+ EVs, indicating that the majority of the hyperoxia-induced EV GSDMD originated in AEC. Moreover, they suggest a novel mechanism for our recent observations that GSDMD activation is a part of the inflammasome pathway activated by hyperoxia in the lung and brain of neonatal mice^22^. To our knowledge, this is the first study reporting increased lung epithelial-derived active GSDMD in circulating EVs in experimental models of BPD. When combined with previous reports of microparticle GSDMD release by activated monocytes in vitro^25^ and GSDMD plasma microparticles in adult septic patients^23^, they suggest that the activation of cellular GSDMD by inflammatory injury and its release in circulating EVs/microparticles may be common to many inflammatory diseases.

While the autocrine-like effect of intracellular inflammasome-activated GSDMD to induce cell death and secondary inflammation is certain^17–19^, whether EV GSDMD can function in a paracrine-like or endocrine-like manner to induce neighboring cell or distant organ injury is currently unclear. However, such bystander signaling mechanisms, although not directly attributed to GSDMD, are well documented for both localized and systemic EV-mediated radiation-induced cellular death^26^. But, our adoptive transfer experiments do suggest that endocrine-like mechanisms are possible as they revealed that intravenously injected RA-EVs and O_2_-EVs were both taken up by the lung and brain tissues of recipient normal neonatal rats. More importantly, we found that adoptive transfer of O_2_-EVs into normal newborn rats produced lung changes similar to those seen in hyperoxia-induced rodent models of BPD^20–22^. These changes were not seen in their counterparts who received RA-EVs. Histologically, the changes seen in O_2_-EVs recipients were decreased alveolarization and vascular density, two hallmarks of BPD, suggesting the adoptive transfer of O_2_-EVs was responsible for the creation of a BPD phenotype in these otherwise normal newborn rats. These histological changes were accompanied by evidence of BPD-characteristic lung inflammation as increased counts for total leukocytes, lymphocytes, macrophages and neutrophils were found in BALF, and increased gene expression of the BPD-associated cytokines IL-18, TNF-α, CXCL1, CTGF and TGF-β1 was found in lung tissue samples. IL-18 and TNF-α are pro-inflammatory cytokines previously reported to be associated with BPD or pulmonary hypertension in preterm infants or experimental models of BPD^27–30^. Interestingly, IL18 is activated by the inflammasome, and its secretion is dependent on GSDMD-p30 pore formation^17–19^. CXCL1 is a pivotal chemokine that regulates neutrophil recruitment during infection^31^ and chronic inflammatory responses characterized by increased CXCL1 and TNF-α have been observed in hyperoxia models of BPD^32^ as well as in neonatal lung injury induced by bromine^33^. While CTGF and TGF-β are fibrosis-inducing cytokines previously shown by us and others to play a causal role in inducing abnormal alveolarization and fibrosis in the developing lungs in general and in hyperoxia-induced rodent models of BPD in particular^34–36^. Taken together, these results suggest that upregulation of these important inflammatory and fibrotic mediators contributes to the induction of BPD pathology by O_2_-EVs in the receipt rats. Although many cell types in the lung could be the targets of circulating EVs, we focused our investigation on PVEC because PVEC would have direct contact with the circulating nanoparticles, and their injury can lead to abnormal vascular development in BPD^37^. Utilizing in vitro PVEC cultures, we demonstrated that O_2_-EVs decreased cell proliferation and increased cell death. We speculate that these in vitro vascular endothelial effects may translate into poor vascular growth as well as endothelial cell damage, leading to vascular leakage that would allow inflammatory cells to infiltrate into the alveolar airspaces, as noted in our in vivo results.

Although it has been long known that BPD is an independent risk factor for adverse neurological outcomes in preterm infants^4–6^, the underlining mechanisms connecting lung injury to brain injury are poorly understood. However, our previous findings show that inflammasome-activated GSDMD plays a critical role in the brain injury seen in a mouse model of BPD^22^, and circulating EVs are known to play a key role in inter-organ communications. Thus, we additionally investigated if adoptive transfer of hyperoxia-activated circulating EVs could induce inflammatory injury in the brain of normal neonatal rats, as they did to the lung. Our investigation yielded pivotal data that support a novel endocrine-like mechanism in which O_2_-EVs induce the same brain inflammatory injury seen in hyperoxia-exposed mice. We first demonstrated that intravenous injection of both labeled RA-EVs and O_2_-EVs can be detected in the brains of recipient rats by in vivo and ex vivo imaging. Furthermore, we detected increased CSF EVs in both RA-EVs and O_2_-EVs injected rats. These results are not surprising since previous reports show that circulating EVs can cross the BBB and are taken up by brain cells^14,15,38^. Next, and more importantly, we discovered that O_2_-EVs are biologically active as they induced brain inflammatory injury. O_2_-EVs activated microglial cells, and this activation was accompanied, as we had seen in the lung, by increased expression of inflammatory mediators, including IL-1α, IL-18, TNF-α, fibronectin and PDGFRβ. Previous studies have shown that IL-1α, IL-18 and TNF-α are important mediators of neonatal inflammatory brain injury^39,40^, and fibronectin has been shown to promote several pro-inflammatory functions of microglia^41,42^. In the brain, PDGFRβ expressing cells are pericytes, the mural cells of blood microvessels, and these cells play an important role in relaying inflammatory signals from the circulatory system to neurons^43^. We further found that O_2_-EVs induced cell death in the SVZ of recipient rats, which with the SGZ are the main sites of postnatal neurogenesis in mammalian brains^44–46^, both of which we previously found to be sites of cell death in hyperoxia-exposed mice^22^. The developing SVZ and SGZ are enriched in NSC that have the ability to proliferate and generate transient neural progenitor cell clusters (NPC), which then further differentiate into astrocytes, oligodendrocytes, and neurons^44–46^. Since the NPC lie in close proximity to blood vessels, and circulating mediators are known to greatly affect their biological functions^47–49^, they would be logical O_2_-EVs targets. Our in vitro results support this idea as both O_2_-EVs and O_2_-MEVs induced cell death in cultured NSC. Moreover, the O_2_-EVs and O_2_-MEVs induced NSC cell death was found to be pyroptotic in nature and thus likely mediated by EV GSDMD-p30. Collectively, these data reveal a novel in vivo paradigm that links hyperoxic lung injury, lung-derived circulating EVs, and inflammatory brain injury together.

Our study has limitations. One of the limitations is that we did not investigate whether other cell types, such as inflammatory cells and vascular endothelial cells contribute to increased circulating EV GSDMD levels seen in our hyperoxia-induced rat model of BPD. Thus, future studies are needed to determine if any of the circulating GSDMD positive EVs found in hyperoxia-injured neonatal rats are of inflammatory cell-derived or endothelial cell-derived. Another limitation of this study is that we did not explore whether the EV-mediated effects we observed on the lung and brain are totally GSDMD-dependent. Thus, we plan to utilize recently described GSDMD knockout mice^50^ and pharmacologic GSDMD inhibitory approaches similar to those we have previously used^22^ to definitively prove that GSDMD is required for circulating EV-induced lung and brain injury in neonatal rats. Alternatively, studies directed at inhibiting EV formation or cellular uptake will also be important in understanding the mechanisms by which EV GSDMD plays a pivotal role in BPD-associated brain injury.

In conclusion, in this study we demonstrate that hyperoxia stimulates AEC to release GSDMD-p30 laden EVs into the circulation of neonatal rats, and that adoptive transfer of these circulating EVs into normal neonatal rats induces the pathological hallmarks of BPD in the lung. More importantly, these EVs are also capable of crossing the BBB and inducing inflammatory brain injury. We speculate that targeting EV trafficking may provide novel strategies to prevent and treat BPD and its associated brain injury in preterm infants.

## Materials and methods

### Materials

Pregnant Sprague-Dawley rats were purchased from Charles River Laboratory (Wilmington, MA). The following antibodies were used for immunostaining, immunofluorescence staining, Western blot analysis and FACS analysis: rabbit anti-GSDMD and mouse anti-AIF-1 from Novusbio (Littleton, CO); rabbit anti-pro-SPC and mouse anti-CD63 from EDMillipore (Temecula, CA); mouse anti-CD9 antibody from ThermoFisher (Waltham, MA); rabbit anti-Ki67 from Abcam (Cambridge, MA); mouse anti-vonWillebrand factor (vWF) from Dako (Carpinteria, CA); rabbit anti-SPC-PE from Biossusa (Woburn, MA). Total Exosome Isolation Kit was obtained from ThermoFisher. Exo-FLOW Exosome Purification Kit and ExoGlow-Vivo EV Labeling Kit were obtained from Systems Biosciences (Palo Alto, CA). The lipophilic fluorescent dye, 1,1′-dioctadecyl-3,3,3′,3′-tetramethylindocarocyanine perchlorate (DiI) was purchased from Sigma (Louis, MO). MLE-12 cells were obtained from ATCC (Manassas, VA), pulmonary vascular endothelial cells (PVEC) were obtained from Lonza (Walkersville, MD), and rat fetal neural stem cells (NSC) were obtained from ThermoFisher. The primers for all the qRT-PCR were obtained from ThermoFisher.

### Animal models

All animal study protocols (16-030, 2016; 19-030-LF, 2019) were approved by the University of Miami Animal Care and Use Committee with pregnant Sprague-Dawley rats and their pups treated according to NIH guidelines for laboratory animals and the ARRIVE guidelines.

#### In vivo experiment 1: neonatal hyperoxia model, EV isolation and characterization

Newborn rats were randomized on postnatal day 1 (P1) into two groups to receive RA or hyperoxia, 85% O_2_ as previously described^21,22^. On P14, pups were anesthetized, blood samples were collected by right ventricular puncture, and plasmas were obtained. The EVs from an equal volume of plasmas from room air-maintained and hyperoxia-exposed rats were isolated using the appropriate Total Exosome Isolation Kit (ThermoFisher) and resuspended in an equal volume of PBS. A 4 µl from each EV sample were analyzed for particle numbers and size distributions by nanoparticle tracking assay using the Nanosight NS300 system (Malvern Instruments, Malvern, UK) as previously described^16^. Nanoparticle concentrations were expressed per ml of plasma. Expression of GSDMD, SPC, CD9 and CD63, in 20 µg of EVs was determined by Western blot analysis as previously described^22^. For FACS analyses 25 µg samples of plasma EVs were captured on anti-tetraspanin (CD9, 63 and 81)-conjugated magnetic beads (Exo-FLOW Exosome Purification Kit, Systems Biosciences), stained sequentially with an anti-GSDMD antibody and a FITC-labeled secondary antibody, followed by an PE-labeled anti-SPC antibody, and then analyzed using a flow cytometer (CytoFLEX, Beckman Coulter, Brea, CA).

#### In vivo experiment 2: tracking of injected EVs

Plasma RA-EVs and O_2_-EVs obtained in Experiment 1 were labeled with non-lipophilic near IR dye (ExoGlow-Vivo EV Labeling Kit) as instructed by the manufacturer (Systems Biosciences)^51^. The labeled EVs (50 µg/sample) or sham-labeled normal saline (NS) negative control were injected via the tail vein into normal neonatal rats on P7. Whole body imaging was done in vivo at 15 min, 1 h and 4 h after injection, and brain and lung tissues were dissected at 4 h for ex vivo imaging using an In Vivo Imaging system (PerkinElmer, Hopkinton, MA). In a separate experiment, RA-EVs and O_2_-EVs were also labeled with DiI dye (Sigma, St. Louis, MO), and these EVs and sham-labeled NS were injected via the tail vein to a different sets of normal neonatal rats at P7. Brain tissues were collected 24 h later and tissue sections were examined by fluorescent microscopy for Dil signals. CSF was collected by tapping the cisternal magna and EVs were isolated from pooled CSF in each condition and analyzed by nanoparticle tracking.

#### In vivo experiment 3: assessment of EV effects

Normal neonatal rats were randomized to receive adoptive transferring of plasma RA-EVs (pooled from 10 animals) or O_2_-EVs (pooled from 10 animals) isolated in Experiment 1 by temporal vein injection at a dose of 50 µg/animal on P3, and again on P7 via tail vein injection. These rats were maintained in room air, and on P17 they were sacrificed, and lung and brain tissues were collected.

### Assessment of lung inflammation

Rat pups were sedated with 1% isoflurane, tracheotomized with a 22-gauge angiocatheter which was secured in place with a suture. For BAL, ice cold normal saline (0.5 ml) was instilled into the airway and gently withdrawn for 4 times. The lavage was repeated four times to recover a total volume of 1.5–2 ml. The cells were stained with trypan blue and total live cell counts were performed with a hemocytometer. Cytospin (Cytospin 2; Shandon, Waltham, MA) slides were prepared from the BALF and were then fixed and stained using Neat Stain Hematology Kit (Polysciences, Inc., Warrington, PA). A total of 100 cells/slide were viewed and counted for differential leukocyte analysis^52^. Gene expression of inflammatory mediators in lung tissues was assessed by qRT-PCR^53^.

### Assessment of lung morphometry and vascularization

RAC was analyzed by identifying 10 terminal respiratory bronchioles under the 10 × magnification on each H&E stained tissue section. The number of distal air sacs that were transected by a line drawn from a terminal respiratory bronchiole to the nearest pleural surface was calculated and RAC was determined as the average number of distal air sacs from each lung tissue sections^22^. To determine vascular density, immunofluorescence staining for vWF, an endothelial marker, was performed. Ten random images were taken under the 20 × magnification on each lung section and the average number of vWF stained vessels (< 50 μm in diameter) was calculated^22^.

### Assessment of brain inflammation and cell death

The presence of neuroinflammatory microglial cells was assessed by immunohistology for AIF-1. Gene expression of inflammatory mediators was assessed by qRT-PCR^53^. Cell death was determined by TUNEL assay^22^.

### In vitro hyperoxia exposure of cultured MLE-12 cells

MLE-12 cells were cultured in room air or hyperoxia (95% O_2_) in media supplemented with 2% EV-free FBS for 48 h. EVs were isolated from supernatant media with a Total Exosome Isolation Kit (ThermofFisher) and analyzed by Nanosight tracking assay and Western blot.

### In vitro assessment of cell proliferation and death

Room air cultured PVEC and NSC were treated for 48 h with RA-MEVs or O_2_-MEVs and cell proliferation was assessed by immunofluorescent staining for Ki67, cell death was determined by TUNEL and pyroptosis was measured by uptake of PI.

### Western blot analysis

EV concentrations were measured by BCA protein assay using a commercial kit from Pierce Biotechnology Inc (Rockford, IL). Total proteins (20 µg/sample) were fractionated by SDS-PAGE on 4–12% Tris–glycine precast gradient gels (ThermoFisher) and then transferred to nitrocellulose membranes (Amersham, Piscataway, NJ). The membranes were incubated overnight at 4 °C with respective primary antibodies and then incubated for 1 h at room temperature with HRP-conjugated secondary antibodies. Antibody bound proteins were detected using ECL chemiluminescence methodology (Amersham). The intensities of protein bands were quantified by Quantity One Imaging Analysis Program (Bio-Rad, Hercules, CA).

### RNA isolation and quantitative qRT-PCR

Total RNA was isolated from frozen lung and brain tissues and treated with DNase to remove possible DNA contamination as described^53^. One µg of total RNA was reverse-transcribed in a 20 µl reaction by using a first-strand cDNA synthesis kit according to supplier’s protocol (Invitrogen). The Real-time qRT-PCR was performed on an ABI Fast 7500 System (Applied Biosystems, Foster City, CA). Each reaction included diluted first-strand cDNA, target gene primers, or 18S rRNA gene primers and master mix containing TaqMan probes according to the supplier’s instruction (Applied Biosystems). qRT-PCR conditions were 95 °C for 10 min, followed by 40 cycles of 95 °C for 15 s and 60 °C for 30 s. RNase-free water was used as a negative control. The expression levels of target genes were normalized to 18S rRNA.

### Statistical analysis

Data were expressed as mean ± SD and comparisons were performed by Student t-test. *P* values < 0.05 were considered statistically significant.

## Supplementary Information

Supplementary Information.
