# Author Correction: Hyperoxia-activated circulating extracellular vesicles induce lung and brain injury in neonatal rats

**DOI:** 10.1038/s41598-021-99450-2

**Published:** 2021-10-06

**Authors:** Anum Ali, Ronald Zambrano, Matthew R. Duncan, Shaoyi Chen, Shihua Luo, Huijun Yuan, Pingping Chen, Merline Benny, Augusto Schmidt, Karen Young, Nadine Kerr, Juan Pablo de Rivero Vaccari, Robert W. Keane, W. Dalton Dietrich, Shu Wu

**Affiliations:** 1 Division of Neonatology and Batchelor Children’s Research Institute, Department of Pediatrics, University of Miami Miller School of Medicine, P. O. Box 016960, Miami, FL 33101 USA; 2 Department of Neurological Surgery, Miami Project to Cure Paralysis, University of Miami Miller School of Medicine, Miami, USA; 3 Department of Physiology and Biophysics, University of Miami Miller School of Medicine, Miami, FL USA

Correction to: *Scientific Reports* 10.1038/s41598-021-87706-w, published online 22 April 2021

The Competing Interests statement in the original version of this Article was incorrect.

“The authors declare no competing interests.”

now reads:

“JPdRV, WDD and RWK are co-founders and managing members of InflamaCORE, LLC and have licensed patents on inflammasome proteins as biomarkers of injury and disease as well as on targeting inflammasome proteins for therapeutic purposes. JPdRV, WDD and RWK are Scientific Advisory Board Members of ZyVersa Therapeutics.”

The original Article has been corrected.

