# Supplementary material for: Hyperoxia-activated circulating extracellular vesicles induce lung and brain injury in neonatal rats
Source: Sci Rep. 2021 Apr 22;11:8791. doi: 10.1038/s41598-021-87706-w (PMC8062626; doi:10.1038/s41598-021-87706-w)
Supplement: Supplementary file 1 — Supplementary Information. [file 41598_2021_87706_MOESM1_ESM.pdf]

Hyperoxia-activated Circulating Extracellular Vesicles Induce Lung and Brain Injury in Neonatal Rats

Anum Ali[1], Ronald Zambrano[1], Matthew R Duncan[1], Shaoyi Chen[1], Shihua Luo[1], Huijun Yuan[1], Pingping Chen[1], Merline Benny[1], Augusto Schmidt[1], Karen Young[1], Nadine Kerr[2,3], Juan Pablo de Rivero Vaccari[2,3], Robert W. Keane[2,3], W. Dalton Dietrich[2], and Shu Wu[1]*

[1]Department of Pediatrics, Division of Neonatology and Batchelor Children's Research Institute

[2]Department of Neurological Surgery, Miami Project to Cure Paralysis

[3]Department of Physiological and Biophysics, University of Miami Miller School of Medicine, Miami, FL, United States

Figure 1G

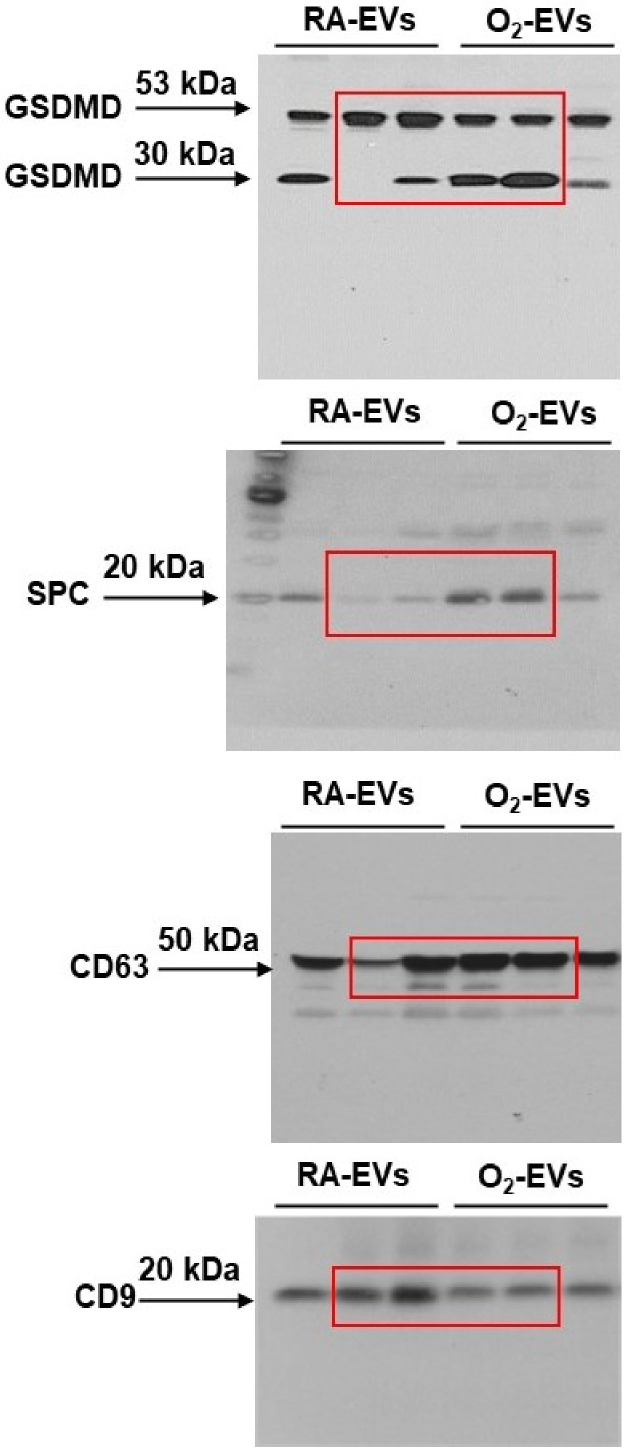
RA-EVs
O2-EVs
GSDMD
53 kDa
GSDMD
30 kDa
RA-EVs
O2-EVs
SPC
20 kDa
RA-EVs
O2-EVs
CD63
50 kDa
RA-EVs
O2-EVs
CD9
20 kDa

Figure 7C

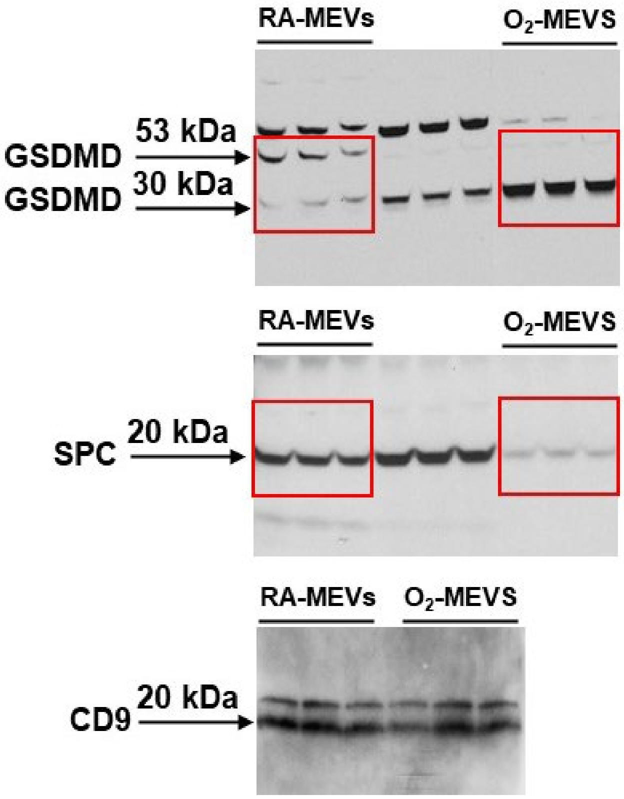

RA-MEVs
O_2-MEVS
53 kDa
GSDMD
30 kDa
GSDMD
RA-MEVs
O_2-MEVS
20 kDa
SPC
RA-MEVs
O_2-MEVS
20 kDa
CD9
